# Value-Added Lager Beer Enriched with Eggplant (*Solanum melongena* L.) Peel Extract

**DOI:** 10.3390/molecules25030731

**Published:** 2020-02-07

**Authors:** Georgiana Horincar, Elena Enachi, Carmen Bolea, Gabriela Râpeanu, Iuliana Aprodu

**Affiliations:** Faculty of Food Science and Engineering, Dunarea de Jos University of Galati, Domnească Street 111, 800201 Galati, Romania; georgiana.parfene@ugal.ro (G.H.); Elena.Ionita@ugal.ro (E.E.); carmen.bolea@ugal.ro (C.B.); gabriela.rapeanu@ugal.ro (G.R.)

**Keywords:** eggplant, beer, bioactive compounds, anthocyanin pigments, phenolic content, antioxidant activity

## Abstract

Manufacturing beer with a high biological value requires identifying new methods for increasing the health-enhancing compounds level. The aim of this study was to increase the biological value of beer by adding antioxidant-rich eggplant (*Solanum melongena* L.) peel extract (EPE). The total phenolic content (TPC), total flavonoid content (TFC), and total monomeric anthocyanin content (TMA) were determined. Moreover, the antioxidant activity was evaluated by different radical scavenging assays. The addition of different levels of EPE resulted in a significant increase of TPC and TFC of beer samples from 0.426 to 0.631 mg GAE/mL, and from 0.065 to 0.171 mg CE/mL, respectively. The EPE-supplemented beer samples developed a reddish color because of the presence of anthocyanin pigments. The TMA content of beer varied from 0.011 to 0.083 mg D3G/mL with the level of added EPE. The HPLC analysis indicated that the anthocyanins prevailing in the eggplant peels were delphinidin-3-rutinoside, delphininidin-3-glucoside and delphinidin-3-rutinoside-5-glucoside. The radical scavenging assays indicated a linear increase of the antioxidant activity following EPE addition, without altering the physicochemical parameters of the beer. These results are promising for using the EPE as a functional ingredient for beer production.

## 1. Introduction

Beer is one of the most appreciated alcoholic beverages worldwide, and has a higher nutritional value than other alcoholic beverages [1]. Beer contains many endogenous antioxidant compounds, mainly arising from malt and hop, among which the phenolic compounds are particularly important for preventing or delaying oxidation processes during brewing and storage of the final product [1]. Because of the natural bioactive compounds found in beer, moderate consumption might provide several benefits for human health, such as a protective cardiovascular effect, control of cholesterol metabolism, blood clotting, and glucose metabolism [2,3]. The mechanisms behind these effects involve the antioxidant compounds from beer [4], which act in different ways, through the complexation of redox-catalytic metal ions, scavenging of free radicals, and decomposition of peroxides [1].

The major antioxidants present in beer include the phenolic compounds and Maillard reaction products [5]. Among these antioxidants, phenolic compounds play a significant role in malting and brewing, due to their ability to delay or prevent oxidation processes [6]. The phenolic compounds identified in beer are mainly represented by phenolic acids, flavonoids, tannins, and amino phenolic compounds [7]. These antioxidants originate from raw materials, such as barley and hop [8]. According to Zhao et al. [6], about 80% of the phenolic compounds present in beer wort are derived from barley malt, and 20% come from hop, all of which are recognized as having important antioxidant and antiradical properties. Some brewing stages, such as mashing and boiling, might affect these compounds, which might even be removed through wort clarification or filtration [9]. Moreover, the degradation of phenolic components might also occur during the fermentation, maturation, bottling, and storage of beer [10]. In addition, during the storage of beer, colloidal haze can form as a result of the interaction between polyphenols, proteins, and polysaccharides [11]. In order to improve the colloidal stability of the final product, beer clarification processes using clarifying agents, such as carrageenan, silica gel, or polyvinylpolypyrrolidone (PVPP), could be applied [8]. Clarification and other technological practices may reduce the content of bioactive compounds and the antioxidant potential of beer. Therefore, in order to produce beers with a high biological value, it is highly desirable to identify new methods for protecting or even increasing the level of health-related compounds in beer.

In the last years, an increasing interest in the valorization of biologically active compounds from food processing by-products has been noticed. Eggplant (*Solanum melogena* L.) peels represent an important source of bioactive compounds, such as anthocyanins, flavonoids, and other phenolic compounds, which provide a myriad of health benefits [12]. In addition to the antioxidant activity, the following functional properties have been reported for whole-plant fruit, pulp, or peel: hepatoprotective [13], anti-inflammatory [14], antiallergic [15], and anticancer activities [16,17]. However, the phytochemical composition and properties of eggplant peel extract (EPE) depend on several factors, such as the plant genotype, climatic conditions, and stage of ripeness at harvest, as well as extraction methods of bioactive compounds [18,19,20].

The aim of the present study was to investigate the suitability of using the EPE for increasing the biological value of beer. The lager beer was supplemented with different levels of EPE and the impact on beer quality was evaluated. In particular, the level of different bioactive compounds, such as anthocyanins, phenolic compounds, and flavonoids, and the antioxidant activity, were monitored over 21 days of storage.

## 2. Results and Discussion

### 2.1. Phytochemical Characterization of EPE

The phytochemical characterization of EPE was performed by an evaluation of the total monomeric anthocyanin content (TMA), total flavonoid content (TFC), total phenolic content (TPC), and antioxidant activity (Table 1). The TMA (1.38 mg D3G/g EPE), TFC (17.06 mg CE/g EPE), and TPC (39.41 mg GAE/g EPE) results obtained for EPE are in agreement with those obtained by Boulekbache-Maklhouf et al. [21] for eggplant peel. They reported a TMA content of about 62.92 mg DGE/100 g DM, a TFC content in the range of 16.26–18.52 mg QE/100 g DE, and TPC within the range of 26.61–29.39 mg GAE/100 g DE.

Todaro et al. [22] extracted anthocyanins and phenolic compounds from fresh eggplant and reported a value of 76.44 mg delphinidin-3-rutinoside equivalent/100 g fresh peel and 188.73 µg GAE/mL extract. Regarding the phenolic composition of eggplant, the predominant compounds are *N*-caffeoylputrescine, 5-caffeoylquinic acid, and 3-acetyl-5-caffeoylquinic acid, while flavonols such as quercetin-3-glucoside, quercetin-3-rhamnoside, and myricetin-3-galactoside are found in trace quantities [23].

Shahidi et al. [24] investigated the anthocyanin profile from eggplant peel and reported that delphinidin-3-(p-coumaroylrutinoside)-5-glucoside (nasunin), delphinidin-3-glucoside, delphinidin 3-glucosyl-rhamnoside, and petunidin are the major anthocyanins in eggplant peel. Our results in terms of the flavonoid content (17.06 mg CE/g EPE) are higher than those reported by Jung et al. [25], who found 6.19 mg CE/g extract from eggplant peels. The differences between our results and those reported in the literature, concerning the content of bioactive compounds from eggplant peels, might be due to the condition of the peel fruit (fresh or dried) or to extraction parameters [26]. Moreover, the influence of genetic and environmental variability should also be considered.

The results of the antioxidant activity of EPE obtained using 2,2-diphenyl-1-picrylhydrazyl (DPPH) and 2,2’-azino-bis-3-ethylbenzothiazoline-6-sulfonic acid (ABTS) methods are given in Table 1. The differences between the antioxidant activities assayed through the DPPH- (0.45 mmol TE/mL) and ABTS (0.032 mmol TE/mL)-based methods might be due to the large influence of the experimental parameters on the reactivity of antioxidants in the samples. In particular, the electron transfer reaction is highly influenced by the chemical structure of the antioxidants, solvent composition, pH of the reaction mixture, and time allowed for the reaction to occur [26]. Moreover, unlike the ABTS-based method, which involves a fast electron transfer process, in the case of the DPPH assay, an addition marginal reaction consisting of hydrogen atom abstraction by DPPH occurs slowly [27]. The electron transfer-based assays quantify the reducing capacity of antioxidant compounds, while the hydrogen atom transfer-based assays measure the hydrogen atom donating capacity.

Jung et al. [25] reported higher antioxidant activity of an eggplant extract in the range of 0.98–1.69 mmol TE/mL. Several studies have shown that the antioxidant activity of vegetal extracts significantly depends on the total phenolic content and other phytochemical compounds, such as proanthocyanins and flavonoids [19,20,21]. However, eggplant represents an important source of bioactive compounds and very few data regarding their utilization in the food industry are available.

### 2.2. HPLC Analysis of Anthocyanins

**Figure 1:**
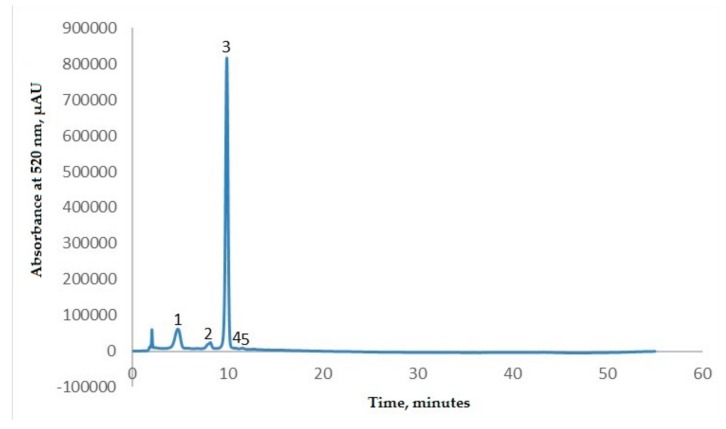
HPLC separation of anthocyanins from the eggplant peel extract monitored at 520 nm. Peak identification: (1) delphinidin-3-rutinoside-5-glucoside; (2) delphinidin-3-glucoside; (3) delphinidin-3-rutinoside; (4) cyanidin-3-rutinoside; (5) petunidin-3-rutinoside.

The HPLC technique was used to identify and find the relative content of anthocyanins from EPE. The identification of anthocyanins from the eggplant peels’ extract was assessed using appropriate standards. The retention time of the standards was considered for assigning peaks in the chromatogram and thus for identifying the anthocyanins present in the sample. The relative content of each anthocyanin was further determined by carrying out a semi-quantitative analysis. The obtained chromatogram (Figure 1) was used for determining the area of each peak as a percentage of the total area of all peaks. According to the results presented in Figure 1, the anthocyanin profile displayed five compounds; delphinidin-3-rutinoside (82.39%), delphinidin-3-glucoside (11.36%), and delphinidin-3-rutinoside-5-glucoside (2.37%) prevailed in the extract, while cyanidin-3-rutinoside and petunidin-3-rutinoside were found at concentrations lower than 0.5% of the total anthocyanin content. Our results are in agreement with those reported in the literature, showing that the major anthocyanin from eggplant peels is delphinidin-3-rutinoside [19,28,29,30].

### 2.3. Influence of EPE Addition on Beer Characteristics

The physicochemical characteristics of beer, such as the alcohol content, extract, CO_2_, and pH, are presented in Table 2. Regardless of the concentration, the addition of EPE to the beer sample resulted in no change of the extract, alcohol content, CO_2_ content, or pH.

Ulloa et al. [8] reported similar results when studying the opportunity to obtain value-added products by the addition of propolis extract in beer. They recorded no changes in the physicochemical characteristics of beer upon propolis extract addition. Moreover, Dordevic et al. [31] studied the possibility of increasing the antioxidant potential of beer by adding medicinal plant extracts and reported that the physicochemical parameters were not affected by the addition of medicinal plant extracts in terms of beer composition. On the other hand, EPE addition caused significant differences in terms of color compared to the control sample, represented by beer without EPE. Because EPE is rich in anthocyanin pigments, the beer samples acquired a reddish color, and the intensity increased with the amount of added EPE. In order to estimate the impact of EPE addition on beer color, the CIELAB color values were monitored over storage (Table 3). The lightness (L*) of the beer samples was measured, together with the chromatic components showing the amounts of green-to-red (a*) and blue-to-yellow (b*). Compared to the European Brewery Convention (EBC) method, the L* a* b* colour analysis was reported to be more efficient when estimating the differences between beer samples [32]. By analysing the results presented in Table 3, it can be observed that EPE addition to beer samples resulted in important changes of the CIELAB colour.

Regardless of the investigated beer sample, all color parameters varied over the entire period of storage. Although a significant decrease of the L* value was noticed after 7 days of storage (*p* < 0.05), the control sample had the highest L* values, ranging from 87.95 to 90.64. A strong greenish shade was measured in the control sample, but a* increased from −2.07 up to −1.17 during storage. Finally, no significant changes of the yellow (b*) contribution to the overall color of control beer was registered over storage.

Increasing the concentration of EPE significantly affected the lightness of beer samples (*p* < 0.05). Furthermore, for samples with EPE over 5 mg/mL, a significant increase of L* values was registered toward the end of the storage period considered in the study (Table 3). As expected, EPE addition to beer resulted in a significant increase of the redness value, and up to 9.86 for the B/EPE 10 sample. This increasing trend of the a* value is a consequence of the higher amounts of anthocyanins found in beer samples with an increasing concentration of EPE. In addition to the increase of redness values, an increase of the yellowness was noticed when increasing the EPE concentration. According to Castaneda-Ovando et al. [33], when the pH of the environment is in the range of 4–6, as is the case of beer samples, the anthocyanins coexist in four different structural forms: a flavylium cation which contributes to purple and red colors, an anhydrous quinoidal base, a carbinol base which is colorless, and pale yellow chalcones. Both redness and yellowness values decreased during storage, most probably as a consequence of the rather low stability of anthocyanins (Table 4).

### 2.4. Phytochemical Characterization of Beer Enriched with EPE

Several studies have reported that different beer processing steps, such as filtration, clarification, boiling, fermentation, and maturation, can produce changes in the bioactive compounds during beer production [9]. In our study, beer was enriched with EPE after the end of the maturation stage, in order to avoid affecting the bioactive compounds through technological processing and to obtain a value-added beer. The phytochemical profile of the beer supplemented with EPE was established by determining the TPC, TMA, and TFC (Table 4).

Anthocyanins are found in nature, mainly in the peel of purple-colored fruits and vegetables, and were not detected in the control beer. In this study, the beer was enriched with anthocyanins by the addition of three different concentrations of EPE (1, 5, and 10 mg/mL). After EPE addition, the TMA content of the beer ranged from 0.011 to 0.083 mg D3G/mL (Table 4). The TMA content was monitored over 21 days of storage under refrigeration conditions, and a significant decrease was registered over the entire tested period for all beer samples supplemented with EPE (*p* < 0.05). This trend can be explained by the fact that TMA are relatively unstable and are susceptible to degradation upon the action of several factors, such as the storage temperature, pH, oxygen, and light access [34]; hence, their concentration decreased until the end of storage. Over the entire storage period, significant linear correlations were identified between TMA and the EPE concentration (R^2^ ranging from 0.9897 to 0.9995 and *p* < 0.005).

The control beer sample had a TFC of 0.065 mg CE/mL and TPC of 0.426 mg GAE/mL, which were rather stable over the entire period of storage (Table 4). Beer contains varying flavonoid amounts, depending on the barley and hop varieties, growing conditions, brewing parameters, and type of beer [35]. These compounds impact the color, taste, flavor, stability, and shelf-life of the beer [35]. The phenolic content of beer depends on the quality and quantity of starting materials and on the brewing parameters, and was shown to highly influence the flavor and colloidal stability of beer.

Beer supplementation with increasing levels of EPE resulted in a linear increase of the TFC and TPC (Table 4). The TFC of beer samples enriched with EPE ranged from 0.075 to 0.171 mg CE/mL, whereas the TPC ranged from 0.439 to 0.631 mg GAE/mL. These results were significantly higher than those of the control sample. Several studies have investigated the presence of bioactive compounds in commercial beer samples. Nardini et al. [36] studied the chemical composition and antioxidant properties of different commercial beers and reported a TFC value ranging from 0.0519 to 0.0732 mg CE/mL. Ulloa et al. [8] evaluated the bioactive compounds from lager beer samples after the incorporation of a propolis extract and reported a TPC increase from 0.242 to 0.307 mg GAE/mL upon the addition of 0.25 g propolis extract/L beer.

Regardless of the EPE addition level, the TFC was rather stable over the entire tested period (Table 4). Although, a slight decrease of TPC was observed after 7 days, at the end of the tested storage period, the TPC of all B/EPE samples (TPC ranging from 0.433 to 0.610 mg GAE/mL) was significantly higher than that of the control sample (TPC of 0.417 mg GAE/mL). Our results are in agreement with those of Li et al. [37], who investigated the stability of phenolic compounds, and reported a TPC decrease of about 18.6% after six months of storage. This behavior was attributed to the oxidation of phenolic compounds by free radicals and polymerization with proteins [38].

### 2.5. Antioxidant Activity

The results regarding the antioxidant potential of beer samples analysed through different methods are shown in Table 5. Regardless of the method used for quantification, the antioxidant activity of beer samples was found to rise with the addition level of EPE, as a result of the presence of increasing amounts of bioactive compounds (Table 4). Similar results were reported by Ulloa et al. [8] and Đorđevic et al. [31], who used different methods to assay the antioxidant activity in lager beer samples after the incorporation of propolis or different extracts of medical plants.

For all tested samples, it was observed that the antioxidant potential exhibited a slight decrease (*p* < 0.05) during storage (Table 5). Although the antioxidant activity of the EPE-supplemented beer samples decreased to different extents over 21 days, at the end of the tested storage period, the antioxidant activity of all B/EPE samples (DPPH radical scavenging activity of 78.047%–79.158%) was significantly higher than that of the control sample (DPPH radical scavenging activity of 56.412%). Higher DPPH radical scavenging activity is important for the stability of beer flavor, because beer staling is generally considered to be associated with the formation of trans-2-nonenal and other saturated and unsaturated aldehydes due to lipid oxidation [7].

The results obtained using the ABTS method are also given in Table 5. As in the case of the DPPH-based measurements, this assay indicated a significant increase in antioxidant activity with an increasing concentration of added EPE. The beer samples with the highest concentration of EPE (B/EPE10 with antioxidant activity of 0.140 mmol TE/mL and 80.019% inhibition) resulted in the highest increase of reducing power compared to the control beer (57.288% inhibition and 0.090 mmol TE/mL). Although the antioxidant activity of samples displayed a slight decrease during storage, the beer samples enriched with EPE had higher antioxidant activity compared to the control sample (without EPE) (Table 5). Our results in terms of radical scavenging activity assays (DPPH and ABTS) are in agreement with other studies. For example, Ullao et al. [8] reported, for beer samples supplemented with different concentrations of propolis extract (0.05, 0.15, and 0.25 g/L), DPPH and ABTS radical scavenging activity values ranging from 0.014 to 0.044 mmol TE/mL and from 0.079 to 0.149 mmol TE/mL, respectively.

## 3. Materials and Methods

### 3.1. Materials

The Pilsner-type malt (Weyermann, Bamberg, Germany) and hop (Pellets 90, Hopsteiner, Mainburg Germany) were used for wort production. The Active Dry Yeast Fermolager^®^ W (AEB, Gretz-Armainvillers, France) was used for the fermentation of lager beer.

Fresh eggplants (*Solanum melongena* L.) of the cultivar Classic harvested in September 2018 were purchased from a local market of Galati, Romania. The eggplant peels were washed with distilled water, dried with paper towels, and then subjected to freeze drying (Christ Alpha 1–4 LD plus, Martin Christ, Osterode am Harz, Germany). Next, the freeze-dried peels were ground into a fine powder by using a laboratory grinder.

All chemicals and solvents used in the study were of analytical grade.

### 3.2. Preparation of the Eggplant Peel Extract (EPE)

The extraction of bioactive compounds from freeze-dried eggplant peels was performed according to the procedure described by Dranca and Oroian [19]. An amount of 1 g of eggplant peel powder was mixed with 20 mL ethanol (70%). The mixture was treated in an ultrasonic water bath (MRC Scientific Instruments, Holon, Israel) at 40 kHz, 40 °C, for 30 min. The samples were then centrifuged at 6500 rpm, at 4 °C, for 10 min. Three successive extraction steps were done, and the resulting supernatant volumes were polled together, followed by concentration to dryness with an AVC 2-18 concentrator (Christ, UK), to get the EPE. In order to be characterized, EPE (1 mg) was further dissolved in 1 mL ethanol 70%. EPE was characterized in terms of TPC, TMA, TFC, and antioxidant activity using the methods described by Turturica et al. [39].

### 3.3. Brewing Process

Beer was produced in a 250 L pilot-scale brewing plant (Dunarea de Jos University of Galati, Romania). An amount of 50 kg Pilsner-type was crushed using a two-roll mill and then transferred into a stainless steel mashing vessel. The infusion mashing process was carried out with a liquor:grist ration of 2.8 L/kg. The process was initiated with the mash-in at 50 °C, and the temperature was then gradually increased and maintained at 64, 72, and 78 °C for maltose production, saccharification, and enzyme inactivation, respectively. After complete mash conversion, the sweet wort was obtained through lautering. The collected wort drained off through the spent grains was directly transferred into the kettle for boiling with the hops. The wort was boiled for 60 min at 100 °C. Two individual hop additions, after 5 min and 50 min of boiling, were performed. The coarse break was separated through settling and the wort was cooled to 11 °C and transferred to the fermentation tank where the brewing yeast was added. The fermentation took place for 7 days at 12 °C, followed by maturation at 0 °C for 14 days. Following maturation, three different concentrations of EPE were added to the beer, consisting of 1, 5, and 10 mg/mL, and were coded as follows: B/EPE 1, B/EPE 5, and B/EPE 10, respectively. The control sample consisted of beer without EPE addition. The quality of the beer samples was monitored over 21 days of storage at 5 ± 1 °C.

### 3.4. Physicochemical Parameters of Beer

The beer samples were characterized in terms of the apparent extract, real extract, alcohol and CO_2_ contents, and color, determined according to ASBC methods beer-3, beer-4, beer-5, beer-10, and beer-13 [40]. The CIELAB color parameters (L*, a*, and b*) were measured using a CR300 Chroma Meter (Konica Minolta) equipped with a D65 Illuminant. The pH was measured by means of a 702SM Titrio pH-meter (Metrohm, Herisau, Switzerland), which was placed directly on the degassed filtered beer samples.

### 3.5. Total Monomeric Anthocyanin Content (TMA)

The TMA was determined using the pH differential method described by Lee et al. [41]. The anthocyanin content was calculated based on delphinidin-3-glucoside, which has a molecular weight of 465 g/mol and an extinction coefficient of 29,000 L/mol/cm (Equation (1)):

Anthocyanins (mg/mL) = A × Mw × DF/ε × L,(1)

where A = [(A_520_ − A_700_)_pH1.0_ − (A_520_ − A_700_)_pH4.5_], Mw = 465 g/mol, ɛ = 29,000 L/mol/cm, DF is the dilution factor, and L is the length of the cuvette (1 cm).

### 3.6. Total Flavonoid Content (TFC)

The TFC was determined using AlCl_3_ solution in methanol, as described by Pai et al. [35]. The results were expressed as mg of catechin equivalents per gram of EPE (mg CE/g EPE) or milliliter of beer (mg CE/mL).

### 3.7. Total Phenolic Content (TPC)

The TPC of EPE and beer samples was determined using the Folin–Ciocalteu spectrophotometric method described by Turturica et al. [39]. The samples were assessed in triplicate and the results were expressed as the mg of gallic acid equivalents per gram of EPE (mg GAE/g EPE) or milliliter of beer (mg GAE/mL). All experiments were done in triplicate.

### 3.8. DPPH Radical Scavenging Activity

The DPPH radical scavenging activity of EPE and beer samples was determined according to the method described by Turturica et al. [39]. The results were expressed as millimoles of Trolox equivalents per milliliter of EPE in ethanol 70% or milliliter of beer (mmol TE/mL). The inhibition percentage was calculated using Equation (2):

% Inhibition = (Ab − As)/Ab × 100),(2)

where Ab is the absorbance of the blank sample (distilled water) and As is the absorbance sample.

### 3.9. ABTS Radical Cation Scavenging Activity

The ABTS radical scavenging activity of the EPE and beer samples was determined according to the method described by Zhao et al. [7]. The results were expressed as mmol TE/mL EPE in ethanol 70% or mmol TE/mL beer. The inhibition percentage was calculated using Equation (3):

% Inhibition = (Ab − As)/Ab × 100,(3)

where Ab is the absorbance blank (distilled water) and As is the absorbance sample.

### 3.10. HPLC Analysis of Anthocyanins

The high-performance liquid chromatographic analysis of the anthocyanins extracted from EPE was assessed with a Thermo Finnigan Surveyor HPLC system with a DAD detector, controlled by the Xcalibur software system (Finnigan Surveyor LC, Thermo Scientific, USA). The method of Turturică et al. [39], with slight modifications, was used. In short, the EPE was filtered through a C18 Sep-Pack cartridge (Cartridge-Waters, USA) to separate the anthocyanins. In order to obtain the chromatographic elution profile, a C18 Synergi 4u Fusion-RP 80A stationary phase column (150 × 4.6 mm, 4 μm) was used, at an optimum column temperature of 25 °C. The mobile phase consisted of two phases: 100% methanol (A) and 10% formic acid (B). The injection volume was 10 μL, at a flow rate of 1 mL/min, whereas the elution took place under the following gradient conditions: 0–20 min, 9%–35% (A); 20–30 min, 35% (A); 30–40 min, 35%–50% (A); and 40–55 min, 50%–9% (A). The samples were filtered through 0.22-μm syringe filters (Bio Basic Canada Inc., ON, Canada) prior to the injection. The detector wavelength was 520 nm. The following HPLC reference substances were used for the identification of anthocyanins: delphininidin-3-glucoside chloride (purity ≥ 95%), delphinidin-3-rutinoside chloride (purity ≥ 95%), and cyanidin-3-rutinoside chloride (purity ≥ 90%) from Sigma Aldrich, Germany.

### 3.11. Statistical Analysis

All analyses were performed in triplicate and the data are presented as the mean and standard deviation. Significant differences among results were identified by means of analysis of variance (ANOVA). Pearson’s correlation coefficients were determined to identify statistical relationships. The one-way ANOVA and Tukey’s test with a 95% confidence interval was applied using Minitab 18 software; *p* < 0.05 was considered to be statistically significant.

## 4. Conclusions

The present study highlights the promising potential of using EPE as a source of bioactive compounds, which allows the antioxidant activity of beer to be increased. The addition of EPE to beer resulted in an increased phenolic content, which is typically affected during the brewing process. In addition to flavonoids, EPE addition provided the beer samples with important amounts of anthocyanins, explaining the reddish color of the sample. The HPLC analysis revealed the presence of five anthocyanins in the EPE, among which delphinidin-3-rutinoside displayed the highest concentration. The amount of anthocyanins decreased over 21 days of storage, whereas the flavonoids displayed a rather good stability. The EPE-supplemented beer has a high functional potential and good sensory characteristics, and is stable without the incorporation of artificial preservatives.

## Figures and Tables

**Table 1:** Bioactive compounds (total monomeric anthocyanins (TMA), total flavonoid content (TFC), total phenolic content (TPC), and antioxidant activities (2,2-diphenyl-1-picrylhydrazyl (DPPH) and 2,2′-azino-bis-3-ethylbenzothiazoline-6-sulfonic acid (ABTS) assays)) for eggplant peel extract (EPE).

**Bioactive Compounds**		
TMA (mg D3G/g EPE)		1.38 ± 0.02
TFC (mg CE/g EPE)		17.06 ± 2.85
TPC (mg GAE/g EPE)		39.41 ± 4.65
**Antioxidant Activity**		
DPPH	Inhibition, %	30.05 ± 0.62
	mmol TE/mL	0.45 ± 0.01
ABTS	Inhibition, %	19.96 ± 0.80
	mmol TE/mL	0.032 ± 0.01

**Table 2:** Physicochemical parameters of the beer control (without EPE) and beer enriched with eggplant peel extract (B/EPE) at different concentrations (1, 5, and 10 mg/mL).

Parameter	Beer Control	Beer Enriched with EPE (mg/mL)		
		B/EPE 1	B/EPE 5	B/EPE 10
Alcohol (% mass)	5.25 ± 0.06	5.20 ± 0.21	5.23 ± 0.56	5.25 ± 0.39
Alcohol (% vol)	6.55 ± 0.03	6.50 ± 0.82	6.52 ± 0.95	6.54 ± 0.23
Real extract (°P)	5.05 ± 0.07	5.01 ± 0.07	5.05 ± 0.07	5.03 ± 0.07
Wort extract (°P)	15.00 ± 0.01	14.80 ± 0.62	15.01 ± 0.04	15.00 ± 0.45
CO_2_ (g/100 mL)	0.46 ± 0.03	0.43 ± 0.29	0.44 ± 0.90	0.45 ± 0.71
pH	4.78 ± 0.04	4.78 ± 0.04	4.78 ± 0.04	4.78 ± 0.04

**Table 3:** Color characteristics of investigated beer samples enriched with different levels of eggplant peel extract (0 mg/mL—control; 1 mg/mL—B/EPE 1; 5 mg/mL—B/EPE 5; 10 mg/mL—B/EPE 10), over 21 days of storage.

Sample	Storage Time, days	Color Parameters		
		L*	A*	B*
Control	0	90.64 ± 0.28	−2.07 ± 0.03	18.47 ± 0.70
	7	89.99 ± 0.70	−1.63 ± 0.05	18.08 ± 1.73
	14	89.65 ± 0.83	−1.35 ± 0.11	18.45 ± 0.50
	21	87.95 ± 0.02	−1.17 ± 0.01	19.10 ± 0.11
B/EPE 1	0	88.00 ± 0.76	−0.80 ± 0.08	19.23 ± 0.72
	7	88.51 ± 0.87	−0.77 ± 0.09	18.12 ± 1.56
	14	87.92 ± 0.96	−0.58 ± 0.11	17.38 ± 1.80
	21	87.86 ± 0.00	−0.47 ± 0.01	15.62 ± 0.08
B/EPE 5	0	81.10 ± 0.81	3.94 ± 0.35	19.59 ± 0.74
	7	81.79 ± 1.06	3.02 ± 0.45	19.67 ± 0.58
	14	84.93 ± 1.06	2.53 ± 0.39	18.37 ± 1.26
	21	86.67 ± 0.08	1.35 ± 0.01	14.66 ± 0.08
B/EPE 10	0	74.67 ± 0.60	9.86 ± 0.35	21.76 ± 0.67
	7	75.44 ± 1.20	8.58 ± 0.32	20.73 ± 0.22
	14	74.51 ± 0.72	8.80 ± 0.37	19.74 ± 0.62
	21	77.56 ± 0.03	7.04 ± 0.02	19.30 ± 0.40

The CIELAB color parameters: L*—lightness; a*—green-to-red; b*—blue-to-yellow.

**Table 4:** Evolution of the bioactive compounds (total monomeric anthocyanins (TMA), total flavonoid content (TFC), and total phenolic content (TPC)) of beer samples enriched with different levels of eggplant peel extract (0 mg/mL––control; 1 mg/mL––B/EPE 1; 5 mg/mL––B/EPE 5; 10 mg/mL––B/EPE 10), during 21 days of storage.

Sample	Storage Time (days)	Bioactive Compounds		
		TMA (mg D3G/mL)	TFC (mg CE/mL)	TPC (mg GAE/mL)
Control	0	nd	0.065 ± 0.006	0.426 ± 0.012
	7	nd	0.065 ± 0.002	0.419 ± 0.000
	14	nd	0.063 ± 0.013	0.416 ± 0.007
	21	nd	0.064 ± 0.004	0.417 ± 0.004
B/EPE 1	0	0.011 ± 0.001	0.075 ± 0.007	0.439 ± 0.013
	7	0.010 ± 0.003	0.076 ± 0.001	0.435 ± 0.001
	14	0.008 ± 0.001	0.074 ± 0.002	0.434 ± 0.005
	21	0.007 ± 0.003	0.070 ± 0.001	0.433 ± 0.007
B/EPE 5	0	0.044 ± 0.002	0.128 ± 0.003	0.544 ± 0.007
	7	0.042 ± 0.001	0.125 ± 0.005	0.525 ± 0.011
	14	0.035 ± 0.001	0.127 ± 0.001	0.523 ± 0.005
	21	0.030 ± 0.001	0.129 ± 0.007	0.519 ± 0.016
B/EPE 10	0	0.083 ± 0.002	0.171 ± 0.009	0.631 ± 0.003
	7	0.073 ± 0.002	0.173 ± 0.001	0.620 ± 0.005
	14	0.071 ± 0.006	0.175 ± 0.001	0.613 ± 0.005
	21	0.068 ± 0.002	0.175 ± 0.003	0.610 ± 0.003

Nd—not detected.

**Table 5:** The antioxidant activity (DPPH and ABTS assays) of beer supplemented with different levels of eggplant peel extract (0 mg/mL—control; 1 mg/mL—B/EPE 1; 5 mg/mL—B/EPE 5; 10 mg/mL—B/EPE 10) over 21 days of storage.

Samples	Storage Time (days)	Antioxidant Activity			
		DDPH (%)	DPPH (mmol TE/mL)	ABTS (%)	ABTS (mmol TE/mL)
Control	0	77.706 ± 0.899	1.200 ± 0.020	57.288 ± 0.371	0.090 ± 0.001
	7	78.502 ± 2.137	1.293 ± 0.041	50.890 ± 2.209	0.089 ± 0.004
	14	59.947 ± 0.191	0.990 ± 0.004	49.867 ± 0.403	0.079 ± 0.001
	21	56.412 ± 1.396	0.926 ± 0.019	49.016 ± 0.521	0.086 ± 0.001
B/EPE 1	0	80.549 ± 0.118	1.244 ± 0.007	58.789 ± 0.405	0.093 ± 0.001
	7	78.685 ± 0.852	1.296 ± 0.017	53.824 ± 2.005	0.095 ± 0.004
	14	78.186 ± 1.111	1.279 ± 0.018	52.178 ± 0.481	0.084 ± 0.001
	21	78.047 ± 0.562	1.287 ± 0.015	53.914 ± 0.413	0.095 ± 0.000
B/EPE 5	0	83.325 ± 0.167	1.294 ± 0.005	73.644 ± 2.643	0.129 ± 0.004
	7	79.396 ± 0.108	1.308 ± 0.004	71.716 ± 0.449	0.124 ± 0.001
	14	78.165 ± 0.558	1.296 ± 0.008	61.144 ± 0.916	0.106 ± 0.002
	21	78.048 ± 0.199	1.287 ± 0.009	57.426 ± 3.096	0.101 ± 0.005
B/EPE 10	0	85.822 ± 0.188	1.333 ± 0.001	80.019 ± 3.167	0.140 ± 0.005
	7	79.839 ± 0.367	1.315 ± 0.012	73.186 ± 0.810	0.126 ± 0.001
	14	79.573 ± 0.309	1.308 ± 0.010	63.685 ± 0.169	0.110 ± 0.000
	21	79.158 ± 0.092	1.306 ± 0.006	60.780 ± 0.653	0.107 ± 0.001
